# Examining the Effect of Overload on the MHealth Application Resistance Behavior of Elderly Users: An SOR Perspective

**DOI:** 10.3390/ijerph17186658

**Published:** 2020-09-12

**Authors:** Yuanyuan Cao, Junjun Li, Xinghong Qin, Baoliang Hu

**Affiliations:** 1 School of Management, Hangzhou Dianzi University, Hangzhou 310038, China; cyy2013@hdu.edu.cn (Y.C.); admire185@126.com (J.L.); bhu@hdu.edu.cn (B.H.); 2 School of Management Science and Engineering, Chongqing Technology and Business University, Chongqing 400067, China; 3 School of Management and Economics, University of Electronic Science and Technology of China, Chengdu 610054, China

**Keywords:** elderly user, mHealth application, overload, technostress, fatigue, resistance behavior, SOR

## Abstract

Aging has increased the burden of social medical care. Mobile health (mHealth) services provide an effective way to alleviate this pressure. However, the actual usage of mHealth services for elderly users is still very low. The extant studies mainly focused on elderly users’ mHealth adoption behavior, but resistance behavior has not been sufficiently explored by previous research. A present study tried to remedy this research gap by examining the effect of overload factors on the mHealth application resistance behavior based on the stimulus-organism-response (SOR) framework. The results indicated that information overload and system feature overload of an mHealth application increased the fatigue and technostress of the elderly user, which further increased their resistance behavior. Meanwhile, we integrated the intergeneration support with the SOR model to identify the buffer factor of the elderly user’s resistance behavior. The results showed that intergenerational support not only directly decrease the elderly user’s mHealth application resistance behavior, but also moderates (weaken) the effects of fatigue and technostress on resistance behavior. The present study also provided several valuable theoretical and practical implications.

## 1. Introduction

The aging of the population has become a prominent worldwide problem. The aging creates great challenges for social healthcare systems. One healthcare study reported that roughly 79.5% of people over 60 years old have at least one chronic disease and almost 50% have more than two chronic diseases [1]. The elderly group is much more likely to suffer from diseases that put pressure on the government and each household to take care of the elderly people. The function of mHealth services facilitates health care for elderly users and brings multiple benefits such as, providing prompting medication alerts, self-monitoring of biometrics indicators, support for telemedicine consultations, health care cost savings, and so on [2,3]. Therefore, mHealth services provide an effective healthcare solution for elderly people. Meanwhile, the design of smartphones has been greatly improved in recent years, and elderly people adopt smartphones at a high rate and rely on them, which provides a golden opportunity to develop mHealth application services for them [1]. However, despite the potential benefits and opportunities for elderly people to use mHealth application services, they are found to be less innovative toward it and encounter various challenges and difficulties when they are using mHealth applications. There are limited elderly users using mHealth application services to manage their health and the actual usage rate is still very low [4,5,6]. This is not conductive to the development of the mHealth industry and alleviates the medical pressures from the aging population. Thus, it is necessary to explore the inhibitor factors which hinder the use of mHealth application services for the elderly people.

Given the benefits of mHealth application services, elderly users’ mHealth usage has received attention from scholars. Theories, such as the technology acceptance model (TAM) as well as the unified theory of acceptance and use of technology (UTAUT) are frequently used as the theory framework [1,6,7,8,9,10]. A variety of influence factors, such as technical factors, personal traits, environment issues, facilitating issues were verified to affect elderly users’ mHealth usage [1,7,8,9,10,11,12,13]. The prior research greatly enriched our understanding of elderly users’ mHealth usage. However, some research gaps still existed. (1) Most of the prior research focused on acceptance behavior [6,7,8,9,10,11]. Fewer research pay attention to the formation mechanism of resistance behavior and how to alleviate and reduce it. (2) Although the researchers have already taken efforts to investigate the various influence factors for elderly user’s mHealth application usage, much less about the overload factor and negative emotional factor. However, these two type inhibiting factors have been found to be the significant influence factors for the information system usage [14,15,16,17,18], particularly for the elderly users who are unfamiliar with information technology products, they will be more affected [19]. These research gaps may hinder our comprehensive understanding of the elderly users’ mHealth application usage and effective preventive measures to facilitate elderly user’s usage. Thus, the present study attempts to address these gaps by examining how the overload problem existed in the mHealth application system lead to elderly users’ negative emotion and further influence resistance behavior.

To achieve the objectives of the present research, an empirical study was conducted in China.

By 2050, China’s elderly population will reach 480 million, accounting for almost a quarter of the world’s elderly population [20]. China is about to become one of the countries with the most serious aging problem. The Chinese government has vigorously promoted the development of mobile healthcare to alleviate the social healthcare pressure caused by aging. Although a large number of mHealth applications are emerging, most elderly people in China have not used them. The utilization rate is still very low [21,22]. This state is consistent with the background of this study. Thus, we carried out the present research based on the case of China.

Stimulus-organism-response (SOR) was employed as the theoretical framework to uncover the elderly users’ mHealth application resistance behavior [23]. We proposed that information overload and system feature overload (stimulus) will trigger elderly users’ fatigue and technostress (organism), which in turn leads to their resistance behavior (response). Meanwhile, in China, filial piety culture demands adult children should support their elderly parents [12,24,25]. Digital feedback from the children has an impact on parents’ attitudes and behaviors to use information technology [26,27,28,29]. Thus, in the present study, intergenerational support as one cultural variable was integrated into the model to examine the direct and mediator buffer effect on the resistance behavior.

The contribution of this study is as follows: first, the present study extends the elderly users’ mHealth application usage research by exploring the resistance behavior. Second, the present study identifies two key overload characteristics in the mHealth application environment and the varying mediator effects of negative emotion, which enriched the prior literature that ignored these factors. Third, our study shows that intergenerational support can serve as a buffer factor to reduce elderly users’ resistance behavior, which provides a solution on how to alleviate the elderly user’s mHealth application resistance behavior. These findings of the formation mechanism of elderly user’s mHealth application resistance behavior are also helpful for managers to take targeted preventive measures.

## 2. Literature Review and Theoretical Background

To provide a comprehensive theoretical research background for the present study, the existing studies related to the elderly user’s mHealth usage were firstly reviewed which is helpful to understand the existing research gaps and the aim of the present study. Then, the SOR was drew upon as the overarching framework, three sets constructs which included information overload and system feature overload, fatigue and technostress, and resistance behavior were respectively integrated into the SOR as the components of stimulus, organism, and response. Intergeneration support was also integrated into the model to explore the buffer factor of the resistance behavior. The brief review of these theories and related studies are provided below, which serve as a theoretical background for the development of the research model and hypothesis.

### 2.1. Elderly User’s MHealth Usage

As an emerging application, mHealth products have not been widely used by elderly users.

Some of the studies found that age was an important factor that affected users’ willingness to use mHealth products. For example, Kaphle et al. (2015) found that the likelihood of lower mHealth adoption rate will increase with age [30]. Leigh et al. (2020) indicated that the increasing patient age and costs resulted in significant reductions in digital health prescription [31]. Accordingly, based on this realistic situation, there is a significant body of elderly users’ mHealth usage research which mainly focused on the adoption behaviors that have sprung up.

UTAUT and TAM are the most frequently used theoretical frameworks in prior research. As an influential theory in the information system adoption context, UTAUT was proposed by Venkatesh, Davis, and Davis (2003) [32]. Three core variables including performance expectancy, effort expectancy, and social influence in the UTAUT are used to predict the user’s adoption attention. TAM is also the most influential model for technology adoption. The TAM proposed that the actual technology use is determined by the technology use intention, whereas in turn, intention can be predicted by the perceived usefulness and perceived ease of use [33]. The existing research mainly combines the specific situation variables and the characteristics of the elderly user to expand these two models to examine the elderly users’ mobile medical adoption behavior. For example, Hoque and Sorwar (2017) integrated UTAUT with resistance to change and technology anxiety to examine the factors that influence the adoption of mHealth products by elderly users [6]. Gmaa et al. (2017) combined UTAUT with perceived credibility to investigate the factors that affect elderly users’ mHealth services using intention [9]. Cimperman et al. (2016) extended the UTAUT with the doctor’s opinion, computer anxiety, and perceived security to analyze elderly users’ home telehealth services acceptance behavior [7]. Hsiao and Tang (2015) integrated TAM with perceived ubiquity, health knowledge, health care need and subjective norm to examine mobile healthcare technology acceptance by elderly users in Taiwan [10]. Guo et al. (2013) constructed an integrated model with TAM and technology anxiety, resistance to change, dispositional resistance to change to reveal the dark side of elderly users’ acceptance of mHealth services in China [8]. In addition to the research based on the technological models of TAM and UTAUT, some studies also explored elderly users’ mHealth behavior from other perspectives. For example, based on the protection motivation theory and social cognitive theory, Fox and Connolly (2018) found that mistrust, high-risk perceptions and a strong desire for privacy affect elderly users’ mHealth adoption intention [11]. Lee et al. (2017) noted that context and contents values have a positive effect on elderly users’ mHealth application use intention [13].

The influence factors on the adoption behavior are various [34], except the mHealth usage research focused on the elderly users, there is also a significant body of mHealth adoption studies from the general public’s perspective which are helpful to comprehensively understand the characteristics of the elderly user. For example, based on the extended TAM, Hoque (2016) found that perceived ease of use, perceived usefulness, and subjective norm had significant positive association with the mHealth service adoption intention among younger people in Bangladesh [35]. To et al. (2019) drew upon the extended TAM to explore Chinese young adults’ mHealth use intention. They found that perceived usefulness had a strongly significant influence on mHealth use intention. Communication effectiveness, health consciousness, and perceived ease of use were found as the significant factors influenced the young people’s intention to use mHealth through perceived usefulness. Meanwhile, it is worth noting that perceived ease of use has direct negative influence on mHealth use intention in the study of To et al. [36]. Alam et al. (2020) integrated UTAUT with privacy, lifestyles, self-efficacy, and trust to explore the influence factors of mHealth application adoption for the young generation. The results confirmed that performance expectancy, social influence, hedonic motivation, and privacy exerted a positive influence on behavioral intention whereas facilitating conditions, self-efficacy, trust, and lifestyle influenced both behavioral intention and actual usage behavior [37].

Meanwhile, some other researchers further explored the differences in mHealth application adoption behavior between elderly users and other age groups. For example, Deng et al. (2014) compared the middle-aged and elderly users’ adoption of mobile health services based on the value attitude behavior model (VAB) and the theory of planned behavior model (TPB) [1]. The results revealed that perceived value, attitude, perceived behavior control, and resistance to change were the main predictors for the middle-age group’s mHealth adoption. Excepting the perceived value and perceived behavior control, technology anxiety, and self-actualization were the other two significant predictors that affected the elderly group’s mHealth application adoption intention. Guo et al. (2016) compared the different effects of privacy-personalization paradox factors on mHealth application acceptance intention between youth and elderly users [38]. The results showed that few elderly users were concerned with privacy as a problem compared with young people.

To sum up, although the existing studies have already explored elderly users’ mHealth usage from multiple perspectives, some research gaps still exist.

Firstly, innovation acceptance and resistance are the two main behavior reactions when users encounter an innovative product [39]. However, the existing studies mainly focused on elderly users’ mHealth adopting behavior. Few studies have paid attention to the elderly users’ mHealth application resistance behaviors, which is a common phenomenon among the elderly users [22]. That will hinder our comprehensive understanding of elderly user’s mHealth usage and adopt efficient interventions.

Secondly, existing studies had already explored some influence factors which will affect elderly users’ mHealth usage mainly based on the TAM and UTAUT frameworks, such as technology factors (e.g., perceived ease of use, perceived useful) [8,10], personal traits (e.g., technology anxiety [1,8], perceived security, perceive physical condition [1], resistance to change [8], self-actualization need [1], health care need, health knowledge [10], privacy concern [11]), environmental issues (e.g., social influence [13], subjective norm [12]), facilitating issues (e.g., availability of support, facilitating conditions [7,9]). Although these influencing factors under the TAM and UTAUT framework can help us indirectly understand the elderly users’ mHealth resistance behavior to a certain extent, the TAM and UTAUT frameworks are mainly used to predict the user’s adoption behavior [32,33]. Adoption and resistance behavior are not two completely equivalent opposite concepts, in other words, non-adoption is not equal to resistance [40]. The influence factors or the constraints from adoption studies cannot fully understand resistance behavior. On the one hand, adoption behavior refers to the acceptance behavior when the user initially contact with information products, which occurred at the beginning of the information system usage life cycle [40]. However, resistance behavior can occur in the whole life cycle of information system usage, such as resistance behavior in the post-adoption stage [41]. On the other hand, the research perspectives for the adoption and resistance are usually different [40]. The former mainly explains the user’s acceptance behavior mainly based on the perception, attitude, and behavior intention [42], while the latter explains the causes of the user’s resistance to the information technology mainly based on the changes or negative influence bring from the system implementation [43]. Therefore, the influence factors under the TAM and UTAUT frameworks cannot fully understand resistance behavior. Direct research on resistance behavior is needed. Moreover, previous studies have shown that system design defects and negative emotions are important reasons for resistance behavior [44,45], but these influence factors were seldom mentioned in the existing elderly user’s mHealth application use research. Thus, the present study tries to fill these research gaps to explore how specific system design defects and negative emotions trigger elderly users’ mHealth resistance behavior.

Thirdly, there are some similarities and differences in mHealth usage between different age groups. For example, perceived usefulness, subjective norm, perceived value, and perceived behavior control are the common influencing factors for both elderly and young mHealth product users [1,8,10,35,36]. Meanwhile, there are some differences between elderly and young users in some aspects, such as privacy issues [32], and technology anxiety [1,6,8]. It is worth noting that for the perception of ease of use, the research conclusions about elderly users are relatively consistent, that is, ease of use has a significant positive effect on adoption intention. However, for young users, the results are mixed. Some studies have found that perceived ease of use may have positive [35] or negative effects [36] for younger users. In other words, for older users, they generally accepted that the mHealth application functions should be simple and easy to use, while for young users, there may be some young users who want more system functions. Thus, for the elderly user and young user, the perception of product design complexity may be different. Due to the age barrier for the elderly user, accordingly, they are more likely to perceive overload factors in the mHealth environment compared with the young user. The perceived overload factors such as perceived system feature overload and information overload reflected the system design defects. Thus, the effect of these two factors on the elderly users’ mHealth application resistance behavior will be examined.

Meanwhile, according to the universal design (UD) principles of product design, “simple and intuitive use”, “perceptible information“, and “low physical effort to decrease the fatigue“ are the core requirements for a product [46], and the design process of mHealth product should avoid the overload system function and information and reduce the perception of negative emotion such as fatigue and technostress. Therefore, through comprehensive consideration of the existing research results and UD principles, the present study will examine the resistance behavior forming mechanism from the perceived overload and negative emotion perspective.

### 2.2. Theoretical Framework of Stimulus-Organism-Response (SOR)

The SOR model is a classic paradigm to explain how external stimulus interacts with an individual’s inner state, which finally leads to an individual’s behavior. This model emphasizes that the stimulation of the individual is caused by the external environment. Under the stimulation of various external factors (stimulus), the individual will make the corresponding behavior changes (response) through the judgment of his inner psychological activities (organism) [23].

The SOR model is a well-suited theory framework in the present study for the following two reasons: firstly, it has been widely used to explore the users’ behavior responses in the information system context. For example, Zhou (2019) examined the relationship among community quality, social support, trust, and users’ knowledge sharing intention in the online health community context based on the SOR model [47]. Fu et al. (2018) examined the effect of external similarity and internal similarity on users’ perceived usefulness, perceived enjoyment, trust toward members, and their subsequent impact on the online purchasing intention of movie tickets [48]. Yao et al. (2017) explored the effect of social interaction overload, invasion of work, and invasion of privacy on perceived usefulness, technostress and perceived enjoyment, which in turn further influence users’ social network rational usage intention [15]. Based on these existing studies, it is confirmed that the SOR model is a suitable overarching framework in explaining an individual’s internal psychological perception and behavior responses in the face of environmental stimuli generated from the information system. Secondly, SOR comprises three main components, namely stimulation, organism, and response to explain the forming process of users’ behavior, which is in line with the propose of the present study, i.e., to explore how overload-related stimulus trigger elderly users’ inner psychological perception, and finally generate the resistance behavior. Thus, the SOR model will be applied to investigate the forming process of elderly users’ mHealth application resistance behavior in this study.

#### 2.2.1. Perceived Overload as Stimulus (S)

Perceived overload refers to an individual’s subjective evaluation and perception of things that exceed a person’s processing capacity. The mismatching of environment demand and personal processing demand leads to overload. According to the technology overload framework proposed by Karr-Wisniewski (2010) [14], system feature overload, information overload, and communication overload were three main salient elements in technology overload. System feature overload refers to the given technology being too complex for a given task. Information overload refers to the information received by the user in the information system exceeding the user’s processing capacity. Communication overload refers to when an individual is interrupted by too much communication which leads to the reduction of work efficiency. Different from the social information system product, the main purpose of using mHealth application for elderly users is to obtain a health information service rather than social or communication. Therefore, based on the present research context, information overload and system feature overload will be considered as the main perceived overload factors in the mHealth system environment.

The prior studies have confirmed that perceived overload was an important factor that caused users’ negative perception, such as fatigue, regret, technostress, emotional exhaustion, and so on, and then further triggered users’ negative behavior [16,18,49,50,51,52]. However, the existing research mainly focuses on social information system products which are mainly used for entertainment, relaxation, socializing, such as WeChat, Facebook, Twitter, Pinterest, and the research objects are mainly young users. For elderly users, will the perceived information overload and system feature overload in the mHealth information system environment generate their negative emotional perception and then further trigger their resistance behavior? How do we alleviate the influence of these negative factors on the elderly users’ mHealth product? These questions need to be further verified.

#### 2.2.2. Fatigue and Technostress as Organism (O)

The SOR model suggests that the user’s inner organism perception plays a mediate role between outside environment stimuli and users’ behavior response [23]. Previous studies in the information system filed confirmed the two crucial roles of user inner organism perception that are fatigue and technostress.

Fatigue refers to a self-assessed unpleasant feeling as a result of psychological and physical factors [53]. Psychological fatigue is characterized as negative perception, such as tiredness, boredom, exhaustion, and so on. Physical fatigue is characterized by a lack of energy, long-term exhaustion, and weakness. These two forms of fatigue may not appear at the same time in every situation. According to the different research contexts, fatigue may appear in one form or both forms at the same time. Physical fatigue usually occurs in a compulsory environment related to physical work rather than the voluntary context [17]. For the present study context, elderly users’ mHealth application using are commonly voluntary behavior, thus, the mHealth application using fatigue can be regarded as a form of psychological fatigue which usually manifests as negative emotional reaction such as tiredness, boredom, burnout and so on [17,54].

Technostress refers to “a stress or psychosomatic illness caused by using technology” [55]. Prior studies have uncovered technostress creators which including workload increase, techno invasion, technical complexity, and difficulties understanding, technology insecurity, technology upgrading too quickly [56]. Technostress has a significant influence on the user’s behavior. Resisting the new technology is one manifestation of technostress [15].

Overload is one of the main negative factors that affects the use of information technology products [14]. Fatigue and technostress are the two significant results of the perceived overload which has been verified in many empirical studies [15,16]. Additionally, fatigue and technostress are also important drivers for the negative use behavior of the information system and play an important mediator role between the relationship of perceived overload and information system negative usage [15]. Thus, the present study attempts to verify whether the fatigue and technostress experience fully or partially mediates the relationship between elderly users’ perceived overload and resistance behavior in the mHealth application context.

#### 2.2.3. Resistance Behavior as Response (R)

User resistance behavior refers to individuals’ adverse reactions and objections to potential changes caused by the implementation of the information system [57]. User resistance behavior occurs at any stage of information system implementation including pre-implementation stage, during-implementation stage, and post-implementation stage [41]. According to the intensity of resistance, the external manifestations of users’ information system resistance behavior can be divided into four levels: apathy, passive resistance, active resistance, and aggressive resistance [58,59]. Specifically, apathy means that the user is not interested in the implementation of the information system and appears to be inactive, estranged, distant, lacking interest, and so on. Passive resistance means that the user influences the implementation of the information system through some implicit behavior, which is manifested as complaining, delaying tactics, persistence to previous behavior, withdrawal, and so on. Active resistance refers to the user taking some obvious but non-destructive actions to obstruct the implementation of the information system, such as publicly expressing inappropriate opposite comments, instigating others to jointly resist the implementation of the new system, and so on. Aggressive resistance refers to the user adopting some destructive actions to prevent the information system implementation, such as strikes, boycotts and sabotage. Active and aggressive information system resistance behavior usually exists in the working environment [58,59]. Based on the present research context, we mainly focused on elderly users’ mHealth application resistance behavior with apathy and passive external character.

Existing studies have explained the information system resistance behavior based on human-oriented theory, system-oriented theory, human–system interaction theory, fairness-implementation theory, expectation theory, status quo preference theory, and so on [40,57]. It has been found that the user’s characteristics, system characteristics, negative emotions, negative expectations, cognitive differences, organizational support, technical or social changes are the important factors that affect user resistance behavior [40]. Different research situations determine the differences in influence factors. An mHealth application is mainly used for individual health information and service acquisition, thus user behavior is mainly affected by system or individual-level factors rather than organizational-level factors. Prior studies found that system design defects, such as the unfriendly interface or complex operation, are the main reasons for user resistance [45]. Meanwhile, the information system implementation can stimulate users’ emotional response. When users experience unpleasant negative emotions such as sadness, anger, pain, disgust, fatigue, stress, and fear, users tend to resist the implementation of an information system [44]. Elderly users usually have poor control over the information system products. In the mHealth environment, due to the aging barriers, such as, declined cognition, perception, and physical abilities [60], the elderly users are more prone to perceived overload from the system function and information feature which will further generate fatigue and technostress. Perceived information overload and system feature overload reflect the defects of system design. Fatigue and technostress reflect the negative emotion. According to the prior studies [44,45], all these factors may be the significant incentives for resistance behavior.

Therefore, this study will explore the influence factors of elderly users’ resistance to mHealth application from the perspective of the perceived overload and negative emotion.

### 2.3. Intergenerational Support

Intergenerational support generally refers to the process of financial reciprocity, mutual help in life, and emotional mutual support between parents and children. Filial piety is a traditional Chinese culture [24]. Children have obligations to help their parents, including respect, care, and support for elderly people [12]. In China, intergenerational support in the opposite direction, that is, providing intergenerational support from children to elders, is a very common and representative cultural feature [25]. With the development of information technology, intergenerational support is also reflected in digital feedback. Support from the children plays an important role in helping the elderly user learn how to use social media, mobile phones and other new information technologies [27]. At the same time, medical resource support from children, such as medical information and care, is important for elderly users to achieve healthy aging [61].

In the present study, intergenerational support refers to the emotional or technology support from children to their elderly parents in using mHealth applications. Based on the above statement, the present study speculates that the intergenerational support from children may help the elderly users to use mHealth application and reduce their resistance behavior towards it. Thus, we will integrate intergenerational support into the SOR theory framework. On the one hand, we will explore the direct effect of intergenerational support on resistance behavior. On the other hand, we will examine the moderate effect of intergenerational support between negative emotion and resistance behavior.

## 3. Research Model and Hypotheses

**Figure 1:**
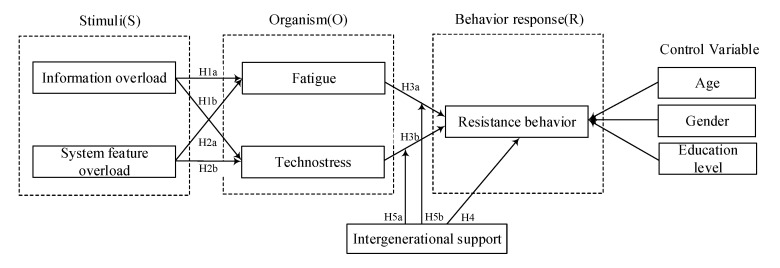
Research model.

Based on the theoretical framework of SOR and the above literature review, the research model was proposed which is shown in Figure 1. Meanwhile, the prior studies found that users’ age, gender, education level influence the information system usage [42]. Thus, we adopt these three variables as control variables.

### 3.1. Overload (S) and Psychological Perception (O), Resistance Behavior (R)

#### 3.1.1. Information Overload and Fatigue, Technostress

Providing health information is the main service for the mHealth application. With the development of mHealth applications, a huge amount of health information was produced and diffused. However, information overload arises when the energy (e.g., cognitive effort and time) which is needed in information processing exceeds the individual’s ability [62]. Processing overloaded information is prone to cause “information fatigue syndrome”, which is reflected as increased anxiety and insomnia symptoms, and fatigue [51]. At the same time, homogenized and low-quality information is also the main cause of information overload which is positively related to fatigue in the information system environment [49]. Repetitive and false information often make users miss the really useful information, or consume a lot of time in distinguishing the authenticity and usefulness of information, which will cause users to have strong irritability [51].

The positive relationship between information overload and fatigue has been validated in prior studies. For example, Lin et al. (2020) indicated that information overload is the main predictor of social media fatigue [51]. Lee et al. (2016) found that information overload as a stressor that will make the user feel overwhelmed and emotional exhaustion [49]. Meanwhile, for elderly users, the declined physiological functions and lower health information literacy will exacerbate the effect of information overload on fatigue. Thus, based on the existing studies, we state that:

**Hypothesis 1a** **(H1a).** *Information overload is positively related to the fatigue for the elderly users*.

Information overload in mHealth applications increases the workload of elderly users. The portable and mobile features of mobile devices limit the interface and content that can be displayed. A large amount of information exists within different page sections or columns in the mHealth application system. It is difficult for elderly users to recognize the icons and understand the functionality of buttons with the interface. Therefore, elderly users are likely to fail to find the relevant information that is only visible after clicking the button [63]. Meanwhile, the font of the information content in the mobile application is usually small. For the elderly user with reduced vision, reading of excessive information will increase their workload [64], which will cause technical pressure. Thus, based on the existing studies, we state that:

**Hypothesis 1b** **(H1b).** *Information overload is positively related to the technostress of elderly users*.

#### 3.1.2. System Feature Overload and Fatigue, Technostress

To attract users and enhance their experience, many system functions are integrated into the mHealth application. Although adding special system functions will make the product more unique and increase efficiency, too many functions may cause overload and make users feel overwhelmed [14,49]. For example, in the online health community, which is mainly used for professional online health consultation, some auxiliary system functions, such as invoice management, medication and rehabilitation diary are also integrated into the system. Too many system functions can easily interfere with the usage of the main function for elderly users. At the same time, the online health community updated frequently, and the new added or improved functions after the upgrade will cause elderly users to be unfamiliar with the new system that will also lead to the function overload. It has been verified by some empirical studies that the system feature overload has a significant positive effect on fatigue [52]. This is in line with the function fatigue theory. When elderly users use an information product, they usually use only a few specific functions according to their purposes. The complexity of product functions increases operation difficulty and interferes with the main functions being used, resulting in functional fatigue [50]. Compared with younger users, the elderly group has a higher level of computer anxiety and less control over information technology products [1]. Thus, when elderly users face complex operations in mHealth applications, they are more likely to feel frustrated and fatigue. Thus, we state that:

**Hypothesis 2a** **(H2a).** *The system feature overload is positively related to the fatigue of the elderly user*.

Most elderly people are not able to operate mobile application proficiently [64]. Function complexity is one of the causes of system feature overload. The complex functions of mHealth applications will take much more time and energy for the elderly user to learn how to operate. The complexity of technology brings increased workload and psychological burden, it will bring technical pressure [49]. Meanwhile, frequent system upgrades and corrections will also bring the system feature overload [49]. MHealth applications usually periodically upgrade the systems, and the uncertainty brought by the frequent upgrade of system versions is one of the reasons for the technical pressure. For elderly users, they are accustomed to familiar things [19], the addition of new functions will cause elderly users to be unfamiliar with the operation interface and unable to find the required function options. Learning new functions is usually a big obstacle [65], which will bring them technical pressure. Thus, we state that:

**Hypothesis 2b** **(H2b).** *The system feature overload is positively related to the technostress of elderly users*.

### 3.2. Psychological Perception (O) and Resistance Behavior (R)

According to the coping model of user adaptation [66], when the user was threatened by technology, they will take an adaptation strategy to avoid or minimize negative outcomes and unpleasant feelings, such as discontinuance use, absenteeism [67], information avoidance [16] and so on. Existing studies have already indicated that psychological stress and fatigue will cause low participation and have a positive effect on users’ resistance activities. For example, Zhang et al. (2016) verified that social network fatigue will reduce the intensity of use and even abandon it completely [17]. Luqman et al. (2017) found that the excessive social use, hedonic use, and cognitive use of Facebook will lead to technostress, which in turn brings discontinuance usage intention [68]. Elderly users will face greater perceptual barriers when they are using mHealth applications [64], which may bring greater negative emotions and technical pressure. According to a coping model of user adaptation, when elderly users experience higher levels of stress and technostress, they are more likely to adopt resistance behavior to escape from negative psychological perception. Thus, we state that:

**Hypothesis 3a** **(H3a).** *Elderly users’ mHealth application fatigue is positively related to resistance behavior*.

**Hypothesis 3b** **(H3b).** *Elderly users’ mHealth application technostress is positively related to resistance behavior*.

### 3.3. The Role of Intergenerational Support

Increasing attention has been paid to children’s technical support for elderly users in recent years [27]. Young groups are ahead of the elderly group in terms of information technology acceptance and usage. Digital feedback provides effective technical support for elderly users’ internet use and promotes their new technologies using [28]. It is found that in the early diffusion stage of new technologies such as computers, mobile phones, and the internet, children are very active in teaching and helping parents master new technologies [29]. Families with children tend to use more digital media. Children usually encourage and explain the method of using information commutation technology to their grandparents [26]. Although the support from the children is only one of the many factors influencing the diffusion and use of new technology, their influence can’t be ignored. Therefore, based on the existing research, we infer that children who are better at using information technology will give some support to an elderly parent to help them solve difficulties in using mHealth products, thus reducing the elderly user’s resistance behavior towards the mHealth application.

**Hypothesis 4** **(H4).** *Intergenerational support reduces elderly users’ mHealth application resistance behavior*.

Age barriers including declining cognition, motivation, physical and perception ability hinder the use of mHealth applications for elderly users [64]. It requires repeated attempts and learning for them to master the mHealth application using methods. The negative emotions such as fatigue and technical pressure formed in this process can easily cause frustration in elderly users and lead them to give up using applications [17]. Previous studies have shown that technology and emotional support from children can promote new technology products using for elderly users [28]. Thus, we state that the more intergenerational support of elderly user obtains in the process of using mHealth application, the more likely it is to mitigate the impact of negative emotions on resistance behavior.

**Hypothesis 5a** **(H5a).** *Intergenerational support moderates (weakens) the effect of fatigue on elderly users’ mHealth application resistance behavior*.

**Hypothesis 5b** **(H5b).** *Intergenerational support moderates (weakens) the effect of technostress on elderly users’ mHealth application resistance behavior*.

### 3.4. The Mediator Role of Fatigue and Technostress

According to the SOR model, the outer environment stimulator will cause behavior responses through the inner states of perceptions [23]. The inner states of perceptions play the mediator role in this process. Prior studies from the social network system environment have verified that overload factors in information systems will cause users’ negative emotional perceptions, which in turn further influence users’ negative behavior. For example, Guo et al. (2020) found that social network fatigue partially mediated the relationship between information, social overload, and information avoidance behavior [16]. Luqman et al. (2017) implied that technostress and exhaustion mediated the relationship between excessive social, hedonic, cognitive use, and discontinuance usage intention [68]. Cao (2018) indicated that communication and social overload will indirectly influence the discontinuous intention through regret in social media [18].

Together H1a, H2a, and H3a suggest that the fatigue which the elderly user felt from the mHealth application will meditate the relationship between information overload, system overload, and elderly user’s resistance behavior. Meanwhile, together with H1b, H2b and H3b suggest that the technostress which the elderly user perceived in the mHealth APP application will meditate the relationship between the information overload, system overload, and elderly users’ resistance behavior. Thus, based on the SOR model and prior studies, we stated that:

**Hypothesis 6a** **(H6a).** *Fatigue mediates the effect of information overload on elderly users’ resistance behavior*.

**Hypothesis 6b** **(H6b).** *Fatigue mediates the effect of system overload on elderly users’ resistance behavior*.

**Hypothesis 6c** **(H6c).** *Technostress mediates the effect of information overload on elderly users’ resistance behavior*.

**Hypothesis 6d** **(H6d).** *Technostress mediates the effect of system overload on elderly users’ resistance behavior*.

## 4. Methodology

To verify the research model and hypotheses proposed in the present study, an empirical study was carried out by the questionnaire survey.

### 4.1. Measurement

The research model included six reflective variables. All the variables were measured with multiple items. The measurement scales used in the present research were adapted from the existing relevant literatures regarding mHealth service, elderly user’s mHealth application usage, information system environment overload, information system resistance behavior, and so on. To ensure consistency, following the back-translation procedures which were widely used in the prior studies [69,70,71], one researcher translated the initial English version of the questionnaire into Chinese. After that, the other three researchers translated them back into English independently as a double check. Meanwhile, to improve the content validity of the measurement, all the items were modified according to the mHealth application research background and the character of elderly users. A five-point Likert scale was used which ranges from 1 to 5 (1 represents “strongly disagree”, 5 represents “strongly agree”). The pretest was conducted on 50 elderly users who resist using the mHealth application. Based on the feedback from the pretest, some improper questions were modified or deleted to ensure the reliability, validity, and understandability of the questionnaire. The final items and reference sources are shown in Table 1.

Items of information overload and system feature overload were adapted from Karr-Wisniewski and Lu (2010) [14]. Items of information overload measure the extent to which the elderly user is exposed to too much information that exceeds their processing ability in mHealth application. Items of system feature overload measure the extent to which the elderly user perceive that the function features provided by mHealth application exceeds their demand and operational capability. Items of fatigue are adapted from Ahsberg (2000) [54], which measure the degree of an elderly user’s subjective negative feelings from using mHealth applications such as tiredness, boredom, burnout, and so on. Items of technostress adapted from Ayyagari et al. (2011) [55], which measure the degree of stress caused by using an mHealth application. Resistance behavior was adapted from Kim (2011) [72], which measures the elderly user’s adverse reactions to the implementation of mHealth application. Intergenerational support was adapted from He and Huang (2020) [73], which measure the support from children to their parents for using mHealth application services.

### 4.2. Sampling Design

The definition of elderly users’ age is various according to the different countries and research background. Since the present research was conducted in China, we defined the age of elderly users as 60 years old and above according to the elderly people’s age definition in the law of the People’s Republic of China on the Protection of the Rights and Interests of Seniors.

An online survey was carried out through Wen Juan Xing, a professional questionnaire platform in China. The respondents were selected according to the following criteria: (1) the participants should be 60 years old and above. They should have mobile devices, such as mobile smartphones or pads, and so on. (2) Participants should have the behavioral characteristics and views of resisting the use of mHealth applications. For example, some elderly users complain or oppose the use of mHealth applications or some elderly people initial install mHealth application, but when they experience it, they feel bad and then uninstall it. Participants are required to give the name of the mHealth application which they have resisted using and then answer the questionnaire according to their use experience and perception. We scrutinized all the responses and dropped those who did not meet the criteria. Finally, 317 valid responses were obtained. The basic demographic of respondents and mHealth product information are shown in Table 2. To display the mHealth application information more clearly, according to the core function of mHealth application and classification standards in the mHealth industry [74], we have classified the mHealth application provided by participants and listed the corresponding specific applications. The mHealth applications covered in the survey included the following main types: online health community which is mainly used for providing professional telemedicine consultations; the doctor appointment mHealth application is mainly used for providing the medical guidance to find the suitable doctors and helping the patient to make an appointment with the doctor online; the medical e-commerce mHealth application is mainly used for providing a drug information inquiry and purchase online.

### 4.3. Common Method Variance Testing

The common method variance testing was conducted by two tests. First, Harman’s single factor test was employed. Six factors were extracted and the largest variance is 15.3%. Thus, the majority of the variance can not be explained by none of an individual factor. Second, according to the method suggested by Rönkkö and Ylitalo [75], three items of social motivation were used as marker variable items. The social motivation had low correlations with other items in our research and collected in the same survey. Then, social motivation as an exogenous variable was included in the model to predict each endogenous construct in the model. Finally, we found that the significant paths in the baseline model were still significant in the method factor model by comparing the baseline and method model. Thus, the common method variance problem will not influence the results of the present study.

## 5. Data Analysis and Result

The data analysis was performed by the structural equation model (SEM). SEM analysis was conducted by four steps. Firstly, the validity and reliability of the measurement model were analyzed. Secondly, the structural model was examined to verify the research hypotheses. Thirdly, the mediation effect test was carried out. Finally, the moderation effect test was performed.

### 5.1. Measurement Model

Smart PLS 2.0., a user-friendly modeling software for partial least squares (PLS) which has been widely adopted in the existing research [69,70], was employed to analyze the measurement model and structural model. The reliability and validity of the measurement model were examined. The values of composite reliability (CR) and average variance extracted (AVE) were assessed to evaluate the reliability of the measurement model. As shown in Table 3, the CR and AVE for each factor were larger than the threshold value (CR > 0.7, AVE > 0.5), suggesting that all the factors in the research model had good reliability.

Convergent validity was examined by the value of factor loadings, AVE, CR, Cronbach’s Alpha (CA). As shown in Table 3, all the indicators were above the standard values (factor loading standard > 0.7, AVE standard > 0.5, CR standard > 0.7, CA standard > 0.7), suggesting that all the convergent validity of the constructs were acceptable. Discriminant validity was examined by comparing the square root of AVE and correlation coefficients. The results are shown in Table 4, the square root of AVE for each factor were larger than its correlations with the other factors, suggesting the discriminant reliability was acceptable.

### 5.2. Structural Model

**Figure 2:**
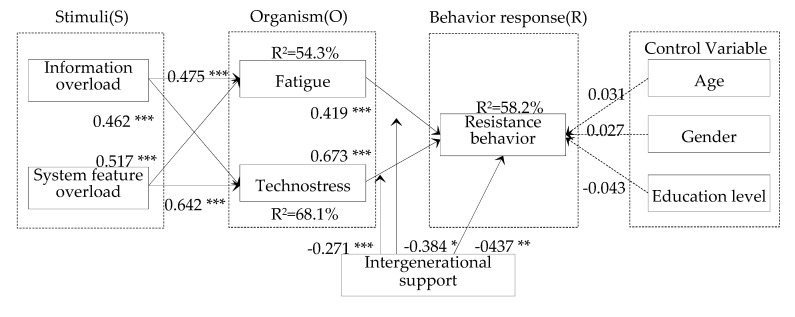
Structural model. (Note: *, *p* < 0.05; **, *p* < 0.01; ***, *p* < 0.001).

The path coefficients and R^2^ of the dependent variables are shown in Figure 2. The results show that both information overload (β = 0.475, *p* < 0.001) and system feature overload (β = 0.462, *p* < 0.01) have significant positive association with fatigue. Thus, H1a and H1b are supported. Meanwhile, information overload (β = 0.517, *p* < 0.001) and system feature overload (β = 0.642, *p* < 0.001) also have significant positive association with technostress. Thus, H2a and H2b are supported. The positive association between fatigue and resistance behavior is significant (β = 0.419, *p* < 0.001). The positive association between technostress and resistance behavior is significant (β = 0.673, *p* < 0.001). Thus, H3a and H3b are supported. The negative association between intergenerational support and resistance behavior is significant (β = −0.437, *p* < 0.01). Thus, H4 is supported. The explained variance of fatigue, technostress, and resistance behavior is 54.3%, 68.1% and 58.2%, respectively. In additional, three control variables have an insignificant effect on resistance behavior.

### 5.3. Mediation Effect Test

The bootstrap method was employed to test the mediation effect of fatigue and technostress. In step one, the significance of the mediation effect is calculated. Following the suggestion of Nitzl, et al. (2016), if the 95% confidence intervals (CIs) contain zero, the effect is insignificant. On the contrary, the effect is significant. The indirect effect is significant to represent the mediation effect exists. The results are listed in Table 5. For example, for the mediation relationship IO→FA→RB (95 % CI [0.052, 0.173]), the CI interval did not contain zero, thus this indirect path is significant, which indicates that fatigue mediates the relationship between information overload and resistance behavior. Therefore, H6a is supported. Similarly, the other three indirect effects are significant. Thus, H6b, H6c, H6d are supported.

In step two, according to the suggestion of Nitzel et al. (2016) [76], the mediation type is accessed. The partial mediation exists under the condition of both indirect effect and the direct effect are significant; A full mediation existing under the condition of indirect effect is significant and the direct effect is insignificant. Thus, fatigue and technostress respectively play a full mediation role between the relationship of information overload and resistance behavior. Meanwhile, fatigue and technostress respectively play a partial mediation role between the relationship of system overload and resistance behavior.

### 5.4. Moderation Effect Test

The PLS product-indicator approach was employed to analyze the moderate effects of intergenerational support. Following the method of prior studies [77], fatigue, technostress (predictor), and intergenerational support (moderator) were respectively multiplied to create the interaction variables (fatigue◊intergenerational support; technostress◊intergenerational support) to predict the resistance behavior via SmartPLS software. The significance of the moderate effect can be confirmed if the interaction effect is meaningful. In this case, the effect of the interaction variable (fatigue◊intergenerational support) on resistance behavior is –0.384, which is significant at *p* < 0.05. The effect of the interaction variable (technostress◊intergenerational support) on resistance behavior is –0.271, which is significant at *p* < 0.001. Thus, H5a and H5b are supported.

**Figure 3:**
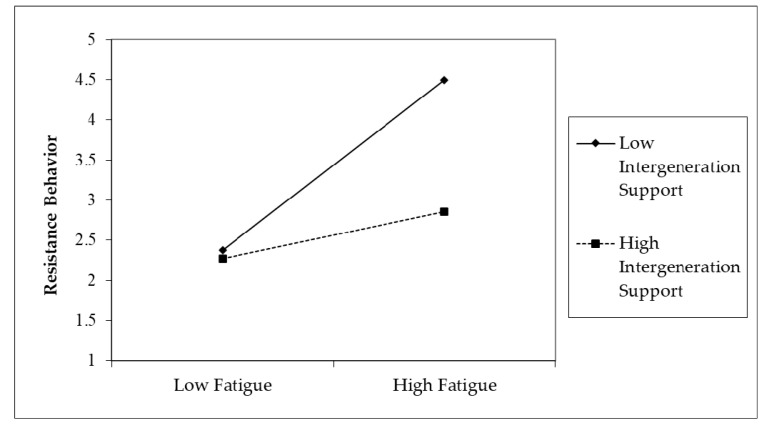
The interaction of fatigue and intergenerational support.

**Figure 4:**
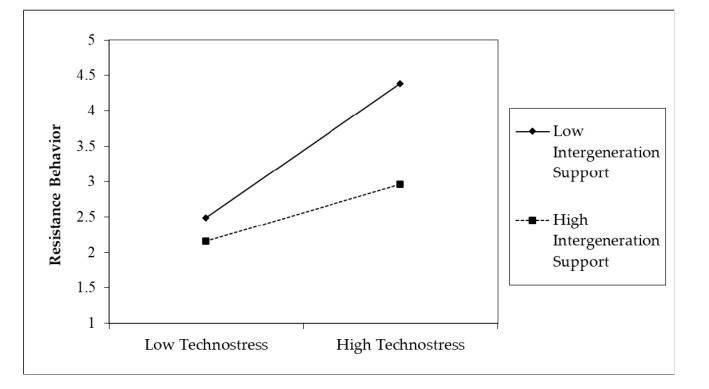
The interaction of technostress and intergenerational support.

The slopes in Figure 3 and Figure 4 indicate that elderly users who perceive a higher level of intergenerational support are less influenced by the effect of fatigue and technostress on resistance behavior than those who perceive a lower level of intergenerational support.

## 6. Discussion

Based on the data analysis and results analyzed in the above section, some key findings provide a fuller and specific understanding of the elderly user’s mHealth resistance behavior. Meanwhile, several noteworthy theory contributions and practical implications are also obtained according to the main result of the present study.

### 6.1. Key Finding

Base on the SOR framework, the present study examines how mHealth application overload factors (information overload and system feature overload) trigger negative psychological factors (fatigue and technostress) and further drive resistance behavior for the elderly user. We also investigate how intergenerational support influences this process. The results provided several key findings as follows.

Firstly, the results indicate that two types of mHealth application stimulus factors which include information overload and system feature overload exert a positive effect on fatigue and technostress for elderly users. These results are in line with prior studies which have verified that the information overload and function overload will increase the users’ negative psychological perception in the information system context, such as fatigue, emotional exhaustion, regret, technostress and so on [18,68]. This phenomenon may be more common in the elderly group due to the age barriers. For example, cognitive aging barriers will cause the reduced ability of attention, comprehension, semantics, and memory [78,79]. The cognitive aging barriers will lead the elderly user to be able to only process much less information in a given time and also forget quickly [64]. Meanwhile, perception barriers, such as vision and audition decline will cause the mHealth application operational difficulties for elderly users [80]. Thus, the enormous amount of information and complex functions in mHealth applications will exceed the coping ability of elderly users and overwhelm them. The elderly users are more likely to perceive fatigue and technostress under the situation of declining health condition.

Secondly, the study examines the role of fatigue and technostress. On the one hand, the result verifies that fatigue and technostress had a significant positive effect on resistance behavior. These results consist of the prior studies which indicate that negative psychological perceptions are crucial predicting factors for users’ information system resistance behavior [17,40]. On the other hand, fatigue and technostress play a mediator role between mHealth application overload factors and resistance behavior. It is worth noting that the type of mediation effect for fatigue and technostress is different in this process. The fatigue and technostress respectively play fully mediator roles in information overload and resistance behavior, which indicates that the effect of information overload to resistance behavior is completely transmitted with the help of fatigue and technostress. However, fatigue and technostress respectively play a partly mediator role in system feature overload and resistance behavior, which imply that a portion effect of system feature overload on resistance behavior is mediated through fatigue and technostress. Meanwhile, system feature overload still explains a portion of resistance behavior that is independent of fatigue and technostress. In other words, when elderly users feel that the system functions are very complex and exceed their capabilities, they may not want to use it or direct give up to avoid fatigue and technostress.

Thirdly, the results of the present study find that intergenerational support has a double role in the formation process of the elderly user’s mHealth application resistance behavior. On the one hand, intergenerational support directly reduces the elderly users’ resistance behavior. On the other hand, we find that intergenerational support moderates (weakens) the effect of fatigue and technostress on resistance behavior. These results are in line with prior studies that suggest that support from children can significantly influence parents’ willingness and behavior to use information technology products [26,28,29]. The results indicate that intergenerational support, such as technical support or encouragement, not only can directly reduce the resistance behavior, but also can weaken the effect of negative emotion on resistance behavior.

### 6.2. Theory Implications

From a theory perspective, the present study contributes to the existing literature as follows.

Firstly, by contrast with most prior studies mainly focused on the positive side of elderly users’ mHealth application adoption behavior [1,6,7,9,10], the present study examines the dark side of mHealth application usage from the resistance behavior perspective. Based on the SOR framework, we investigate how the overload factors (stimulus) influence the inner psychological perception (organism), and further affect the resistance behavior (response). By doing so, it extends the prior research that stops at the adoption usage behavior and complements relevant elderly mHealth using research.

Secondly, this study contrasts with the prior studies which mainly focused on the effect of technical factors, personal traits, environment issues, and facilitating issues on elderly user’s mHealth usage [1,7,8,9,10,11,12,13]. The present study enriches the existed literature by identifying two key overload characteristics (i.e., information and system feature overload) in the mHealth application environment. The empirical results indicate that both two overload factors are important predictors for elderly user’s resistance behavior.

Thirdly, the present study contributes to the existing research by investigating the elderly user’s negative internal psychological perception which is ignored in the prior studies. Our study provides a more comprehensive understanding by examining the varying mediator effects of fatigue and technostress between the overload factors and resistance behavior. The influence of negative emotions should not be ignored in the information system usage environment [17,40]. This notion is supported by the result of the present empirical study which indicates that both fatigue and technostress play critical direct antecedents and mediator role in the formation process of mHealth application resistance behavior for the elderly user.

Finally, this paper explores the mitigating factors that can reduce the elderly user’s mHealth application resistance behavior by integrating the SOR framework with intergenerational support. The result shows that intergenerational support not only directly reduced elderly mHealth application resistance behavior but also indirectly buffers the effect of fatigue and technostress on resistance behavior. Thus, this attempt opens the door to including similar buffering factors to explore how to reduce negative mHealth usage for the elderly user.

### 6.3. Practical Contributions

The findings of this study provide notable practical implications for mHealth service provider.

(1) Information and system feature overload of mHealth applications are two main predictors of fatigue and technostress for the elderly user. Thus, the mHealth manager should pay attention to the overload factor to avoid negative emotion perception and behavior.

On the one hand, the mHealth application managers need to provide higher quality and more personalized information to meet the needs of elderly users; for example, strengthening the information filtering function of the mHealth system, raising the threshold of information release, and reducing the duplicate or contradictory information in the system. The studies on the case-based reasoning system [81,82,83] and healthcare big data technology [84] in the online health field from Gu et al., (2017a,b; 2019; 2020) provided valuable theoretical contribution and practical enlightenment. Meanwhile, in response to the common problems in the elderly health information services, such as “information isolated island”, “data fragmentation” and so on, the research on data-driven organization and decision-making of elderly health knowledge from Gu (2020) provides effective solutions [84]. These research results not only can optimize personalized health information services for elderly user from the microscopic view, but also can meet the information service needs of government departments for the elderly chronic diseases management and decision-making from the macroscopic view. Thus, applying the methods and viewpoints introduced in these studies to the design and management of the mHealth application are helpful to avoid the information overload problem and improve information quality.

One the other hand, the design of an mHealth application should be simple and easy to understand for the elderly user. Designers should remove redundant functions with low usage rates for elderly users and avoid frequent system updates. Adequate functional descriptions and instructions should be organized for the elderly user which is helpful to reduce the technostress and resistance behaviors caused by unfamiliar system functions.

(2) The findings indicate that the effect of fatigue and technostress on resistance behavior can be reduced by intergenerational support. Meanwhile, intergenerational support can also directly reduce resistance behavior. The mHealth application manager can take advantage of this insight and attract children to help their elderly parent to use mHealth application. On the one hand, the manager can do some advertising to emphasize the favorable and valuable aspects for children to help their elderly parents on using mHealth application. Making the children realize the meaningful and necessary to use the mHealth products for their parents’ health care. On the other hand, mHealth developers can design related accounts between elderly parents and their children to make it easier for children to help their parents solve the difficulties or complete corresponding operations in using mHealth application.

## 7. Conclusions

The usage of mHealth applications is very low for elderly users [4,5,6]. Perceived overload factors in the mHealth system and negative emotions are the two significant predictors for elderly user’s mHealth application resistance behavior. Based on the framework of SOR, the present study found that perceived information overload and perceived system feature overload (stimulus) will trigger the elderly user’s fatigue and technostress (organism), which in turn, further lead to the resistance behavior (response). Meanwhile, intergenerational support plays a significant buffer role between negative emotion and resistance behavior. Although the results of the present study provide useful theoretical and practical implications, there are still some limitations. Meanwhile, the future research suggestions are provided as follows.

### 7.1. Limitations

The present study still has some limitations as follows: first, we only examined the main overload factors in the mHealth application context. Other influence factors may still exist to cause negative emotions and behavioral responses. Other possible influence factors need to be furtherly validated based on an integrated research model. Second, the present study was conducted in China. Due to the different culture, the effect of intergenerational support may have different effects in other countries. The generalizability of this finding for intergenerational support should further be verified. Third, the mHealth products involved in the present empirical study are mainly focused on the following types: the online health community, the doctor appointment mHealth application; and the medical e-commerce mHealth application. There are still some other mHealth application types which are not involved in the present empirical study. Thus, whether the findings are still robust for other mHealth types need further verification.

### 7.2. Future Research Suggestions

Accordingly, there are some suggestions to address these limitations for further research. First, a future study can further examine the possible effects of other influential factors on resistance behavior, such as interaction overload, computer anxiety, and so on. Second, the research model can be replicated examined in other countries that have a different culture. In this way, we can compare the buffer effect of intergenerational support on elderly mHealth resistance behavior in the different cultural environment and verify the generalizability of the findings. Third, further study can extend the scope of mHealth application types in the empirical investigation. In this way, we can compare the effect of perceived overload factors and negative emotion on the resistance behavior between different mHealth applications and verify whether the conclusion of this study can be applied to other types of mHealth product.

## Figures and Tables

**Table 1:** Variable measurement and source.

Construct	Measurement Items	Sources
Information overload (IO)	IO1: I am often distracted by the excessive amount of information in mHealth APP	[14]
	IO2: I feel that I am overwhelmed by too much health information in mHealth APP	
	IO3: Processing too much health information is a burden for me	
System feature overload (SO)	SO1: I feel distracted by many features included in mHealth APP which are not related to my main purpose	[14]
	SO2: Some features in mHealth APP are too complex for me	
	SO3: Too many poor sub features in mHealth APP makes performing my task even harder	
Fatigue (FA)	FA1: I feel exhausted during using mHealth APP	[54]
	FA2: I feel boredom from using mHealth APP	
	FA3: I feel drained when I use mHealth APP to search health information	
Technostress (TE)	TE1: The functions in mHealth APP are too complicated and beyond my ability	[55]
	TE2: I feel tired for spending a long time to understand and use mHealth APP	
	TE3: Learning how to operate mHealth APP makes me feel stressed	
Resistance behavior (RB)	RB1: I object to using mHealth APP	[72]
	RB2: I disagree with the using of mHealth software	
	RB3: I oppose the life changes brought by the mHealth APP	
Intergenerational support (IS)	IS1: My children often encourage me to use mHealth APP	[73]
	IS2: My children often instruct me to use some functions of mHealth APP	
	IS3: My children will help me solve the difficulties in using mHealth APP	

(Note: APP: application).

**Table 2:** Demographic of respondents and mHealth product information.

Profile	Sample Composition	Frequency	Percentage
Gender	Male	185	58.36%
	Female	132	41.64%
Age	60–65	196	61.83%
	66–70	113	35.65%
	71–75	7	2.21%
	Over 75	1	0.31%
Education background	Senior High School/lower	197	62.15%
	College	107	33.75%
	Graduate school and above	13	4.10%
Occupation	Public service or educational	79	24.92%
	Information Industry	33	10.41%
	Peasants	68	21.45%
	Retiree	137	43.22%
mHealth applications	Online health community (e.g.,: Chunyu Doctor, Haodafu Online, Dinxiang Doctor, WeDoctor, Pingan Good Doctor)	101	31.86%
	The doctor appointment mHealth application (e.g.,: Wing Health, WeChat Appointment System; Qu Hospital)	73	23.03%
	The medical e-commerce mHealth application (e.g.,: Ali Health, Self-testing Drug, Kangaiduo Palm Drug Store, One Medicine Network)	143	45.11%

**Table 3:** Item loadings, average variance extracted (AVE), composite reliability (CR) and Cronbach’s Alpha (CA) values.

Construct	Indicator	Factor Loading	AVE	Composite Reliability	Cronbach’s Alpha
Information overload (IO)	IO1	0.881	0.798	0.922	0.889
	IO2	0.906			
	IO3	0.892			
System feature overload (SO)	SO1	0.855	0.731	0.891	0.981
	SO2	0.842			
	SO3	0.867			
Fatigue (FA)	FA1	0.861	0.819	0.931	0.809
	FA2	0.938			
	FA3	0.914			
Technostress (TE)	TE1	0.844	0.724	0.887	0.972
	TE2	0.898			
	TE3	0.809			
Resistance behavior (RB)	RB1	0.883	0.755	0.903	0.807
	RB2	0.859			
	RB3	0.865			
Intergenerational support (IS)	IS1	0.944	0.884	0.958	0.912
	IS2	0.935			
	IS3	0.942			

**Table 4:** Latent variable correlation matrix: discriminant validity.

Construct	1	2	3	4	5	6
1. IO	0.893					
2. SQ	0.317	0.855				
3. FA	0.281	0.43	0.905			
4. TE	0.143	0.45	0.352	0.851		
5. RB	0.155	0.19	0.286	0.273	0.869	
6. IS	0.163	0.201	0.374	0.284	0.174	0.940

**Table 5:** Bootstrapped confidence intervals (CIs) mediation test.

Mediation Relationship	Indirect Effect		Direct Effect		Mediation Effect
	95% CIs of the Indirect Effect	Significance or Not	95% CIs of the Direct Effect	Significance or Not	
H6a: IO→FA→RB	[0.052, 0.173]	Yes	[−0.042, 0.134]	No	full
H6b: SO→FA→RB	[0.023, 0.256]	Yes	[0.06, 0.14]	Yes	partial
H6c: IO→TE→RB	[0.037, 0.251]	Yes	[−0.38, 0,71]	No	full
H6d: SO→TE→RB	[0.045, 0.248]	Yes	[0.034, 0.262]	Yes	partial
